# Elevated Flt3L Predicts Long-Term Survival in Patients with High-Grade Gastroenteropancreatic Neuroendocrine Neoplasms

**DOI:** 10.3390/cancers13174463

**Published:** 2021-09-04

**Authors:** Katharina M. Detjen, Raik Otto, Yvonne Giesecke, Lukas Geisler, Pamela Riemer, Henning Jann, Carsten Grötzinger, Christine Sers, Andreas Pascher, Tom Lüdde, Ulf Leser, Bertram Wiedenmann, Michael Sigal, Frank Tacke, Christoph Roderburg, Linda Hammerich

**Affiliations:** 1 Department of Hepatology and Gastroenterology, Charité Universitätsmedizin Berlin, 13353 Berlin, Germany; katharina.detjen@charite.de (K.M.D.); yvonne.giesecke@charite.de (Y.G.); lukas.geisler@charite.de (L.G.); henning.jann@charite.de (H.J.); carsten.groetzinger@charite.de (C.G.); bertram.wiedenmann@charite.de (B.W.); michael.sigal@charite.de (M.S.); frank.tacke@charite.de (F.T.); 2 Knowledge Management in Bioinformatics, Institute for Computer Science, Humboldt-Universität zu Berlin, 12489 Berlin, Germany; raik.otto@hu-berlin.de (R.O.); leser@informatik.hu-berlin.de (U.L.); 3 Institute of Pathology, Charité–Universitätsmedizin Berlin, Charitéplatz 1, 10117 Berlin, Germany; pamela.riemer@charite.de (P.R.); christine.sers@charite.de (C.S.); 4 German Cancer Consortium (DKTK), Partner Site Berlin, German Cancer Research Center (DKFZ), 69120 Heidelberg, Germany; 5 Department of Surgery, Charité Universitätsmedizin Berlin, 13353 Berlin, Germany; andreas.pascher@ukmuenster.de; 6 Clinic for Gastroenterology, Hepatology and Infectious Diseases, University Hospital Düsseldorf, Medical Faculty of Heinrich Heine, University Düsseldorf, 40225 Düsseldorf, Germany; tom.luedde@med.uni-duesseldorf.de; 7 Max Delbrück Center for Molecular Medicine, Berlin Institute for Medical Systems Biology (BIMSB), 10115 Berlin, Germany

**Keywords:** neuroendocrine neoplasm, immuno-oncology, tumor microenvironment, circulating biomarker, Flt3L, cytokine

## Abstract

**Simple Summary:**

Neuroendocrine tumors of the gastrointestinal tract (GEP-NEN) are a rare type of tumor with considerable variability in the course of disease, which makes clinical management particularly challenging. Biomarkers that are able to guide personalized treatment decisions would be of importance, but are not yet available. In this study, we demonstrate that tissue expression, as well as circulating levels of the cytokine Flt3L in advanced and aggressive tumors, predict survival and time to progression. Increased tumoral Flt3L was also associated with upregulation of genes related to immune activation, suggesting Flt3L as a surrogate marker of host anti-tumor immunity. Therefore, Flt3L measurements in serum may hold promise as a biomarker of disease outcome that could support personalized treatment decisions in GEP-NEN patients, potentially guiding researchers towards viable immunotherapies.

**Abstract:**

Background: The clinical management of high-grade gastroenteropancreatic neuroendocrine neoplasms (GEP-NEN) is challenging due to disease heterogeneity, illustrating the need for reliable biomarkers facilitating patient stratification and guiding treatment decisions. FMS-like tyrosine kinase 3 ligand (Flt3L) is emerging as a prognostic or predictive surrogate marker of host tumoral immune response and might enable the stratification of patients with otherwise comparable tumor features. Methods: We evaluated Flt3L gene expression in tumor tissue as well as circulating Flt3L levels as potential biomarkers in a cohort of 54 patients with GEP-NEN. Results: We detected a prominent induction of Flt3L gene expression in individual G2 and G3 NEN, but not in G1 neuroendocrine tumors (NET). Flt3L mRNA expression levels in tumor tissue predicted the disease-related survival of patients with highly proliferative G2 and G3 NEN more accurately than the conventional criteria of grading or NEC/NET differentiation. High level Flt3L mRNA expression was associated with the increased expression of genes related to immunogenic cell death, lymphocyte effector function and dendritic cell maturation, suggesting a less tolerogenic (more proinflammatory) phenotype of tumors with Flt3L induction. Importantly, circulating levels of Flt3L were also elevated in high grade NEN and correlated with patients’ progression-free and disease-related survival, thereby reflecting the results observed in tumor tissue. Conclusions: We propose Flt3L as a prognostic biomarker for high grade GEP-NEN, harnessing its potential as a marker of an inflammatory tumor microenvironment. Flt3L measurements in serum, which can be easily be incorporated into clinical routine, should be further evaluated to guide patient stratification and treatment decisions.

## 1. Introduction

Gastroenteropancreatic neuroendocrine neoplasms (GEP-NEN) are a rare and heterogeneous family of tumors, defined by the production of neuroendocrine vesicular marker proteins such as chromogranin or synaptophysin. GEP-NEN arise from the disseminated neuroendocrine system of the gastrointestinal tract and pancreas and are classified according to their proliferation in a three-tiered grading system, with Ki67 fractions of < 3% in grade 1 (G1), 3% to 20% in grade 2 (G2) and >20% in grade 3 (G3) NEN [1,2]. Further subclassification depends on the morphologic differentiation, which discriminates well-differentiated tumors (NET) from poorly differentiated carcinomas (NEC).

Clinical management of NEN is challenging since the course of the disease exhibits considerable variance in patients with seemingly comparable tumor characteristics. Thus, the survival of patients with advanced stage IV, highly proliferative G2 or G3 NET may range from a few months to more than ten years [2,3]. Even within the group of poorly differentiated NEC, individual patients exhibit unexpectedly slow progression or long-lasting remission after chemotherapy for metastatic disease, with survival times exceeding 5 years. This variability highlights the need for reliable biomarkers that can facilitate patient stratification and guide treatment decisions.

A clinical hallmark of well-differentiated NEN is their extraordinarily dense and highly functional vasculature [4], which implies a very distinct tumor microenvironment that sets NEN apart from most other malignancies [5]. Given the intricate interdependence of angiogenesis and immune surveillance (reviewed in [6,7]), an important contribution of the immune microenvironment in NEN is conceivable even though these tumors feature a low mutational load [8]. Evidence for a major impact of the NEN immune microenvironment on the human disease is also emerging: transcriptome profiles of NEN metastases revealed an enrichment of immune-related signatures when compared to primary tumors [9] and suggested an overall immunosuppressive microenvironment in more advanced or aggressive NEN [10]. In line with this, a recent immune-profiling effort found little evidence for an activation of the adaptive immune system, while a number of genes with known immunosuppressive function were expressed in more advanced G3 NET and NEC [11]. Though differences in tumor immune control are well-known determinants of prognosis and treatment success in a broad range of cancers, few studies have so far addressed the prognostic impact of the immune microenvironment in NEN. Early on, an association between higher numbers of tumor infiltrating CD3^+^ T cells and extended progression free survival was noted in resected intermediate grade pancreatic NET (panNET), while—conversely—a higher number of FoxP3 reactive regulatory T cells in resected liver metastases correlated to worse overall survival [12]. A negative impact on the clinical course of panNET was also proposed for tumor-associated macrophages (TAM) based on their positive correlation with poor disease-specific and disease-free survival [13,14] in G1 and G2 tumors. Recent immunohistological profiling of tumor microenvironment and tumor inflammatory features pointed towards an even more prominent role of immune-related features in high grade NEN [15,16]. In a NEN cohort featuring 40% G3 specimens the authors reported a notable shift of immune tumor microenvironment (TME) marker profiles in G3 compared to G1 and G2 samples, which could be harnessed for improved stratification of the prognostic patient subgroups. In a follow-up study with an extended panel of myeloid and lymphoid markers, the marker-based clustering of samples separated further prognostic subgroups within G3 NEN, with better representation of lymphoid and myeloid markers in the favorable “hot” cluster when compared to the “cold” cluster [16]. Overall, a decisive contribution of adaptive and innate immune responses in more aggressive and advanced disease situations emerges as a common theme, despite the vastly different methodological approaches and NEN cohort compositions, providing a growing rationale for immune-directed therapies. While much-anticipated first clinical trials with immune checkpoint inhibitors in NEN have been disappointing [17], subgroup analyses have suggested more favorable response profiles in high grade NEN [18,19]. Furthermore, a growing number of case reports suggest that individual patients benefit substantially [20,21,22], creating an urgent need to better understand NEN immune control and evasion, especially in patients with advanced aggressive disease, who have otherwise run out of established therapeutic options [23].

Fms-like tyrosine kinase 3 ligand (Flt3L) is a crucial growth factor in the development of conventional and plasmacytoid dendritic cells (DC) and induces the expansion of mature DCs in the periphery [24,25]. Flt3L has been shown to increase the number of circulating DCs in cancer patients [26,27] and has been linked to a favorable clinical course in a variety of cancers [28,29,30]. Conventional type 1 DCs (cDC1), which are highly specialized in antigen cross-presentation to T cells, are the main Flt3L-dependent DC subtype and accordingly, Flt3L abundance in the tumor microenvironment correlates with the presence of cDC1 and the stimulation of effector T cells resulting in improved tumor immune control and clinical outcome in different types of cancers [31,32]. In human melanoma tissues, Flt3L gene expression served as a surrogate marker for cDC1 cell abundance and survival. cDC1 cell abundance in turn correlated with anti-PD-1 responsiveness [31]. In addition, there is evidence from preclinical studies that systemic Flt3L can sensitize cancers to immunotherapies, including checkpoint inhibitors [30,33]. However, no data on the role of Flt3L as a prognostic or predictive marker in GEP-NEN are available.

Here, we studied Flt3L mRNA expression in the tumor tissue as well as circulating Flt3L levels in clinically well annotated cohorts of patients with advanced NEN of pancreatic or gastrointestinal origin. Data were correlated with clinical patient’s characteristics in order to establish a role of Flt3L as a clinically relevant marker in GEP-NET.

## 2. Materials and Methods

### 2.1. Patient Serum Samples

Serum samples were collected from individuals with histologically confirmed diagnosis of pancreatic or gastric NEN or NE-differentiated CUP treated at the Charité Universitätsmedizin Berlin ENETS Center of Excellence (Berlin, Germany), between 2002 and 2017. Sample inclusion criteria were a documented Ki67 index ≥ 10% at any time during the clinical course of the disease and the availability of informative clinical records. Detailed cohort characteristics are provided in Table 1 of the manuscript. Clinical parameters were retrieved from systematic review of the medical records. The study was approved by the local ethics committee at Charité Universitätsmedizin Berlin, Germany (ethical approval number EA1/229/17) and patient informed consent was obtained. Healthy controls were blood donors without medical history of malignant disease aged from 23 to 51 years.

### 2.2. NEN Transcriptome Data

Unpublished RNAseq data from human GEP-NEN with ki67 ≥ 10%, including G3 NET and NEC samples were made available for analysis by Carsten Grötzinger, Charité (manuscript in preparation, raw data available at the EGA database, EGAD00001006657). Data from these Charité samples were then combined with published, publicly available RNAseq data from a panNET cohort that comprised almost exclusively G1 and G2 samples, resulting in a sample set representing the full range of NEN grades and differentiation. From this combined dataset, we used exclusively the data from foregut-derived pancreatic and gastric samples. The resultant sample set focused on panNEN, but 5 gastric G3 NEN were included to obtain adequate representation of the exceedingly rare G3 tissues. A few samples of NEN-adjacent normal pancreas or liver tissue were also available from the Charité dataset and were used as a benchmark for prototypic healthy tissue (which helped to identify and exclude NEN samples suspected of low tumor content and substantial contamination with surrounding normal tissue). The study was approved by the local ethics committee and informed consent obtained from patients that were alive at the time of study initiation. Data obtained from archival samples of deceased individuals was anonymized prior to analyses, restricting the follow-up data to existing clinical annotations.

### 2.3. Determination of Circulating Flt3L

Fresh frozen aliquots of patient or control sera, which had been stored at −80 °C and had not undergone repeated freeze–thaw cycles, were used for measurements of the circulating cytokine. Initial determinations of Flt3L in serum samples were performed using the Human Flt-3 Ligand Quantikine^®^ELISA Kit (R&D Systems, Inc., Minneapolis, MS, USA) according to the manufacturer´s protocol. Absorbance was measured on a Spectramax M plate reader (Molecular Devices, LLC. San Jose, CA, USA), and a log/log curve-fit was used to calculate serum concentrations from the standard curve. Determinations were carried out in duplicate unless low available serum quantity precluded double determination. Values in healthy control samples were below 100 pg/mL, consistent with the reference range provided by the manufacturer.

For subsequent determinations, we switched to the Biolegend Human Stem Cell multiplex bead assay, which allowed the simultaneous determination of 11 additional cytokines in the same quantity of serum. Samples were processed exactly according to the manufacturer´s protocol and measurements of bead-bound fluorescence were carried out on a Becton Dickinson Canto II flow cytometer. Two replicate measurements were conducted for all samples. For interassay normalization we used NEN rather than control samples in order to cover the full range of Flt3L concentrations in NEN, which exceeded the range observed in healthy controls.

### 2.4. Software and Statistical Analyses

Data analysis was performed using IBM SPSS Statistics for Windows version 27.0 (IBM Corp., Armonk, NY, USA) and GraphPad Prism version 7.0 (GraphPad Software Inc., San Diego, California, USA). For tissue-based analyses of mRNA expression, log2 transformed and normalized TPM counts were used. Correlation between mRNA expression of individual genes was assessed with Spearman ρ. Disease-related survival was calculated from the time of blood sampling to the date of NEN-related death (event) or end of follow-up (censored). Progression-free survival was calculated from the time of blood sampling to subsequent documentation of disease progression, including patients with stable or progressive disease at baseline, but excluding tumor-free patients that had undergone curative resection. Survival in patient groups with high or low levels of circulating of FLT3LG protein or tissue-based FLT3LG mRNA expression was compared using the Log-rank (Mantel-Cox) test. Associations between the various NEN-related clinical variables and PFS were modelled with univariate Cox-regression analysis and multivariate Cox-regression, respectively and the results were expressed as hazard ratio (HR) with 95% confidence interval (CI). For comparison of continuous variables between different groups, nonparametric Mann–Whitney U test or Kruskall–Wallis tests were employed, unless stated otherwise. For comparison of categorical variables, Chi-square and Fisher’s exact tests were applied. All reported *p* values are for two-sided tests and considered significant at *p* < 0.05.

## 3. Results

### 3.1. Flt3L mRNA Expression Is Increased in a Subset of G3 NEN

To explore Flt3L mRNA expression in GEP-NEN at the transcriptome level, we identified publicly available datasets from 29 patients that could be used for transcriptomic analysis [8]. In addition, we obtained RNA-sequencing data of 25 samples from GEP-NEN patients at our center (“Charité cohort”). The combined dataset thus encompassed all grades and included poorly differentiated NEN, setting it apart from available published datasets that deliberately focused on G1 and G2 tumors. The cohort (Table 2 and Supplemental Table S1) consisted primarily of pancreatic samples; however, we integrated available G3 samples of gastric origin to obtain sufficient subgroup size for analyses in rarely resected G3 NEN.

**Figure 1:**
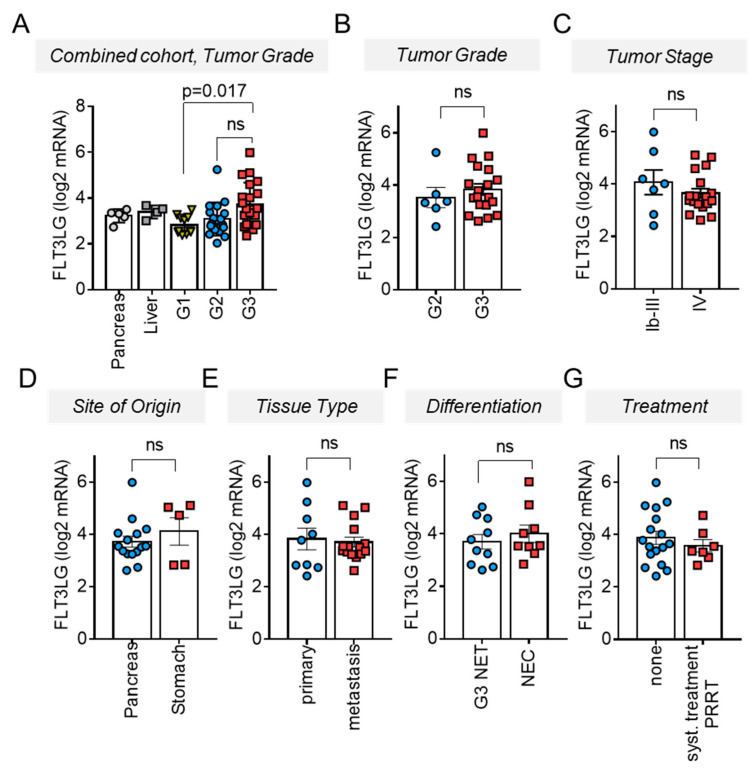
Increased FLT3LG mRNA expression in G3 NEN. FLT3LG mRNA expression data as determined based on the log2 transformed normalized TPM values from the transcriptome data of the combined cohort. Bars and error bars indicate Mean ± SEM. (**A**) Comparison between tumors of different grades revealed increased expression in G3 NEN (*n* = 21) when compared to G1 tumors (*n* = 14) (Kruskall–Wallis test). FLT3LG mRNA expression in non-transformed pancreas (*n* = 5) and tumor free liver tissue (*n* = 4) is shown for comparison. (**B**–**E**) Correlation of FLT3LG mRNA expression with clinicopathological parameters in the advanced tumors of the Charité subcohort. Grades 2 versus 3 (**B**), stage IV (**C**), different tissue origin (**D**), tissue type (**E**), morphologic tumor differentiation (**F**) or prior systemic treatments except SSA (chemotherapy, targeted therapies, PRRT) (**G**) were not reflected by differences in FLT3LG mRNA. Kruskall–Wallis (**A**) and Mann–Whitney test (**C**–**G**) were used for determination of *p*-values; *p* > 0.05 was considered not significant (ns).

In G1 panNET samples, both Flt3L expression level and homogeneity were similar to healthy tissue samples from pancreas or liver (Figure 1A), suggesting a balanced, physiological immune microenvironment in G1 panNET. When comparing tumor samples of different grades, Flt3L mRNA expression in G3 NEN was increased compared to G1 samples and spread over a broad range, with expression in individual samples overlapping with the low expression level present in G1 tumors.

In order to analyze Flt3L mRNA induction in G3 tumor samples, we restricted our analyses to the Charité subset of samples, for which detailed clinical follow-up information was available. We investigated whether Flt3L mRNA expression levels might reflect specific disease characteristics such as stage, site of origin, tissue type (i.e., primary tumor or metastasis), morphological differentiation, or treatments preceding tissue sampling (Figure 1B–G). However, no differences in Flt3L mRNA expression became apparent when comparing G2 and G3 (Figure 1B) or early vs. advanced tumor samples (Figure 1C). Similarly, Flt3L expression levels did not differ between patients with NEN of pancreatic or gastric origin (Figure 1D), primary tumor tissues vs. tumor metastases (Figure 1E) and G3-NET vs. NEC (Figure 1F). Finally, tumoral Flt3L mRNA levels of patients that had already received a tumor specific treatment were not different from levels in therapy-naïve tumor tissues (Figure 1G).

### 3.2. Flt3L mRNA Expression Predicts Disease-Related Survival in Pancreatic and Gastric High Grade NEN

**Figure 2:**
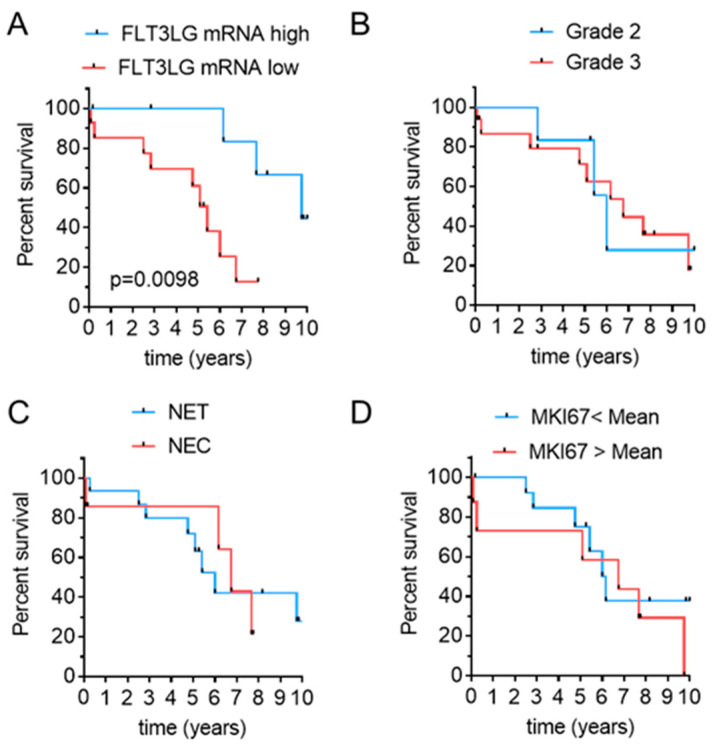
High FLT3LG mRNA expression predicts better survival. (**A**) Kaplan–Meier curves for estimation of disease-related survival in patients with high (log2 FLT3LG mRNA > 3.72, blue) or low (log2 FLT3LG mRNA < 3.72, red) tumoral FLT3LG expression. Follow-up was restricted to 10 years. Median survival was 117 months in FLT3L “high” compared to 65 months in FLT3LG “low“ samples, Hazard Ratio (HR) 0.2404, 95% confidence interval (CI) 0.0716 to 0.8073, *n* = 22. (**B**–**D**) Kaplan–Meier curves for the same cohort following stratification according to tumor grade (**B**), morphological differentiation (i.e., well differentiated NET versus poorly differentiated NEC) (**C**), or MKI67 mRNA expression obtained from the RNAseq data (**D**). Median survival was 72 months in G2 versus 82 months in G3 samples (HR1.007, 95%CI 0.266 to 3.804), 72 months in NET versus 81 months in NEC (HR0.799, 95% CI 0.223 to 2.855), and 81 months compared to 74 months in tumors with MKI67 mRNA above or below mean (HR 1.51, 95% CI 0.476 to 4.808). *p* > 0.05 for (**B**–**D**).

Since we observed a high variability of Flt3L expression in G3 tumors, we asked whether Flt3L mRNA expression might reflect patients´ survival. To establish an ideal cut-off value for the discrimination between survivors and non-survivors, we performed receiver operating characteristic (ROC) analyses. These indicated log2 Flt3L mRNA > 3.72 as best cut-off at 6 and 7 years, respectively (area under the curve (AUC) 0.93 and 0.83) which was closest to the median disease-related survival (DRS) of the Charité cohort. When applying this cut-off for sample stratification, high Flt3L expression levels identified a group of patients with better prognosis (Figure 2A). In contrast, established prognostic parameters such as grading or morphological differentiation did not distinguish prognostic subgroups (Figure 2B–C). Similarly, a separation of these samples based on the mean Ki67 mRNA (MKI67) expression failed to resolve prognostic subgroups (Figure 2D). Thus, Flt3L expression levels emerged as a novel prognostic marker in highly proliferative NEN.

**Figure 3:**
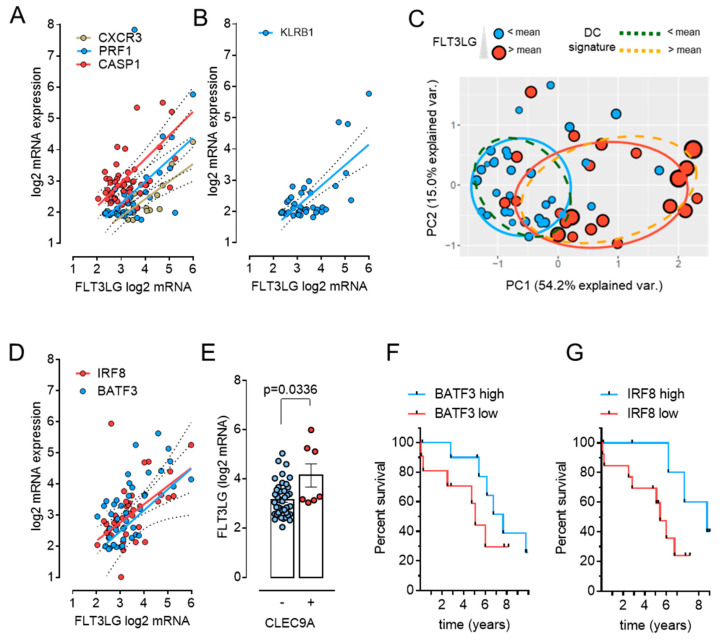
FLT3LG mRNA expression correlates with markers of immunogenic cell death and cDC1 maturation. Correlation of FLT3LG mRNA with mRNA expression of markers for immunogenic cell death (**A**), T- or NK cells (**B**), cDC1 cell maturation (**D**,**E**), and with clustering of samples according to a dendritic cell signature (**C**). Solid and dotted lines in graphs represent linear regression and error, respectively. Correlations were evaluated based on Spearman’s rank correlation coefficient. Tissues with mRNA transcript levels for a given marker at background level were excluded from the correlation analysis, resulting in different sample numbers in analyses for different markers, as is indicated for each marker below. (**A**) Positive correlation of FLT3LG mRNA with three markers of immunogenic cell death (rs = 0.33, *p* = 0.019 for CASP1, *n* = 49; rs = 0.46, *p* = 0.0018 for PRF1, *n* = 43; rs = 0.63, *p* = 0.0181 for CXCR3, *n* = 14). Inclusion of normal tissues allowed an evaluation of FLT3LG correlation with CXCR3 in additional 5 samples and corroborated a positive correlation (not shown, rs = 0.63, *p* = 0.004). (**B**) Correlation with KLRB1 mRNA, a marker present on NK as well as T effector cells; rs = 0.54, *p* = 0.0002, *n* = 44. (**C**) Principal component analysis of the correlation matrix of NEN transcriptomes following supervised clustering based on a gene signature for activated dendritic cells [36]. Symbol colors denote above or below average mRNA expression of FLT3LG (red and blue). Symbol size gives a more gradual indication of FLT3LG expression. Ellipses demark the region of high (dashed yellow) or low (dashed green) representation of dendritic signature genes, and of high (red) and low (blue) FLT3LG expression. Samples with above mean FLT3LG expression mostly project onto the cluster outlined by the dashed yellow ellipse, which features an above mean expression of the signature genes. Note that regions of high FLT3LG and dendritic signature gene expression are almost superimposable. (**D**) Correlation with markers of cDC1 cells IRF8 (rs = 0.55, *p* < 0.0001) and BATF3 mRNA (rs = 0.63, *p* < 0.0001), *n* = 49 for both. (**E**) Comparison of FLT3LG mRNA in tumor samples with (*n* = 7) or without (*n* = 42) detectable mRNA expression of CLEC9A, a receptor specifically expressed on cDC1 dendritic cell subsets. (**F**,**G**) BATF3 and IRF8 cDC1 marker mRNA and disease-related survival. Kaplan–Meier curves of DRS in patients harboring tumors with high (log2 TPM > 3.619, blue) or low (log2 TPM ≤ 3.619 red) BATF3 mRNA (**F**), or with high (log2 TPM > 3.494, blue) and low (log2 TPM ≤ 3.494, red) IRF8 mRNA (**G**). Cut-off values were determined based on ROC curves at 6 years in order to match the FLT3LG analyses.

In order to address the mechanistic basis of the association between Flt3L tissue levels and DRS, we analyzed the association of Flt3L mRNA with expression of marker genes that reflect proposed Flt3L modes of action. Since Flt3L has been linked to immune control and a less immunosuppressive tumor microenvironment via its effects on cross-presenting DC subsets, we specifically tested the relationship of Flt3L mRNA with surrogate markers of immunogenic cell death, NK and effector T-cell function, and specific markers of cDC1 maturation (Figure 3A–D).

Intriguingly, Flt3L mRNA correlated to the markers of a metagene signature indicative of immunogenic cancer cell death [34], which encompassed Caspase1 (CASP1), Perforin 1 (PRF1) and Chemokine receptor CXCR3 (CXCR3) (Figure 3A). Moreover, a positive correlation was obtained for Killer cell lectinlike receptor B1 (KLRB1) (Figure 3B), a T-cell and NK-cell marker gene associated with favorable prognosis across a broad variety of cancers [35].

As cDC1 are perceived as the main Flt3L-responsive cells in the tumor microenvironment, we used the activated dendritic cell gene signature from the well-established LM22 leukocyte gene signature matrix [36] for supervised clustering of the NEN transcriptomes. A principal component analysis based on the correlation matrix separates samples, which exhibit above or below average expression of the dendritic cell signature as indicated by the orange and green ellipse demarcation, respectively (Figure 3C). Of note, most samples with above mean FLT3LG expression (red symbols) reside in the cluster that is enriched for the dendritic cell signature (right cluster). Indeed, the ellipses delineating the projection areas for samples with above (red) or below (blue) average FLT3LG expression are almost superimposable onto those delineating high (orange, dashed line) and low (green, dashed line) DC signature gene expression, consistent with a higher complement of intratumoral dendritic cells in tumors with high FLT3LG mRNA content. Similarly, a correlation matrix heatmap of the NEN samples using a gene signature for intratumoral dendritic cell content [37] supports a stronger representation of the signature in samples with high FLT3LG expression (Supplemental Figure S1A). Most importantly, a positive correlation was identified with mRNA expression of two transcription factors that direct cDC1 differentiation: Interferon Regulatory Factor 8 (IRF8) and Basic Leucine Zipper ATF-Like Transcription Factor 3 (BATF3) (Figure 3D). Furthermore, NEN samples with detectable levels of *CLEC9A* (C-Type Lectin Domain Containing 9A), a receptor expressed on cDC1, exhibited higher expression of FLT3LG than NEN samples, in which CLEC9A mRNA expression was absent (Figure 3E). Of note, high Flt3L mRNA expression levels were noted over a wider range of the overall immune score of samples as determined based on the ESTIMATE algorithm (Supplemental Figure S1B), suggesting it may reflect specific immune cell populations rather than the overall immune cell compartment in individual samples.

Given the excellent correlation of Flt3L mRNA with IRF8 and BATF3, we divided our cohort according to these DC marker genes and determined the relation of IRF8 and BATF3 with DRS in our cohort. Even though these analyses did not reach statistical significance, we still noted a separation of Kaplan–Meier curves, supporting the notion of higher tumoral dendritic cell content underlying better DRS (Figure 3 F–G).

We also analyzed the relation of Flt3L mRNA expression with the neuroendocrine markers synaptophysin (SYP) and chromogranin A (CHGA) (Supplemental Figure S1C) as well as endothelial markers, since Flt3L expression has been reported in endothelial cells. However, neither the neuroendocrine markers nor the endothelial marker genes TIE1 (Tyrosine Kinase with Immunoglobulinlike and EGF-like Domains 1) or CDH5 (Cadherin 5) were significantly associated with Flt3L mRNA abundance in our cohort (Supplemental Figure S1D), while Pecam1 (Platelet endothelial cell adhesion molecule) was weakly associated.

Together, these findings were consistent with a concept of Flt3L abundance as a determinant of tumoral cDC1 content, which in turn offers a mechanistic explanation for improved disease-related survival.

### 3.3. Circulating Levels of Flt3L Are Elevated in Highly Proliferative NEN

**Figure 4:**
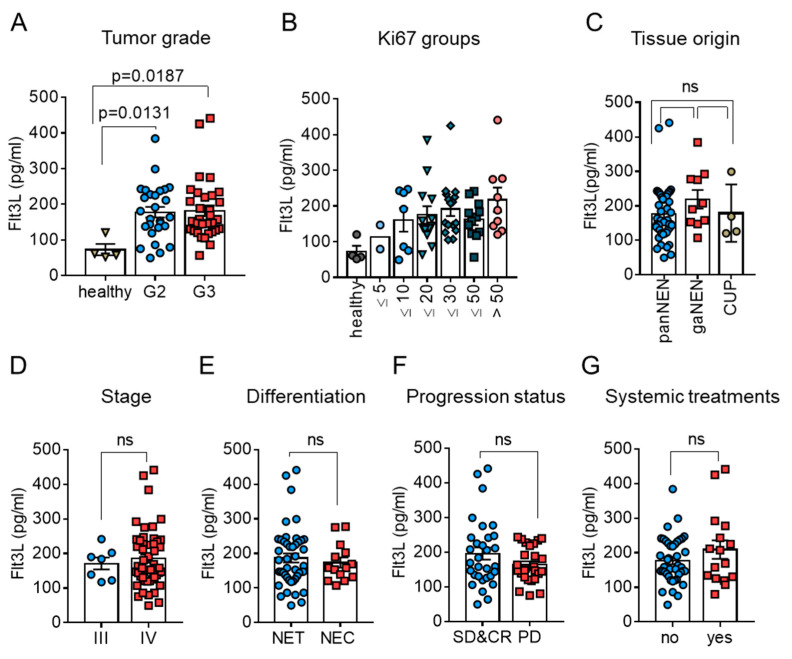
Relation of circulating Flt3L to established clinical features of NEN. Relation between circulating Flt3L and tumor grade (**A**) or ki67 fractions available from clinical records (**B**). Grading and ki67 information were matched as best as possible to the time of blood sampling. Control refers to healthy donor samples (*n* = 4). Bars and error bars in (**A**–**E**) indicate mean ± SEM. Different tissue origin (**C**), presence or absence of metastases (**D**), or morphologic tumor differentiation (**E**) did not translate into differences in circulating Flt3L. Relation to progression status (**F**) and impact of ongoing systemic treatment (**G**). Samples from patients with stable disease (SD) or complete remission (CR) were combined and compared to samples from patients with progressive disease (PD). Systemic treatments included chemotherapies, targeted therapies and PRRT. Kruskall–Wallis (**A**) and Mann–Whitney tests (**C**–**G**) were used for determination of *p*-values; *p* > 0.05 was considered not significant (ns).

Given the encouraging results for Flt3L as a tissue-based biomarker, we hypothesized that the circulating levels of Flt3L might similarly reflect the prognosis of patients with NEN. Therefore, we measured circulating Flt3L levels in a cohort of advanced, highly proliferative NEN (Table 1), matching the main characteristics of the Charité tissue cohort, as well as in healthy controls. In these analyses, Flt3L concentrations ranging from 52 to 128 pg/mL were observed in the control samples (Figure 4A, *n* = 4, referred to as controls), which is in agreement with previously published data [38].

Consistent with the tissue mRNA expression data, a considerable variability of circulating Flt3L levels was detected in samples from patients with high grade NEN (Figure 4A). Since our cohort of patients with high-grade NEN reflected a wide range of proliferative activity in the corresponding tumor tissues, we further subdivided the samples into Ki67 groups, using 10% or 20% increments (Figure 4B) in order to obtain a better resolution of the proliferative activity. This did not uncover significant differences between the Ki67 subgroups, but rather illustrated that substantial variability persisted through the entire range of Ki67 indices.

We therefore determined whether clinical features other than grading/proliferation accounted for the variability in circulating Flt3L. However, logistic regression analyses indicated that neither tissue origin of the primary, nor the stage, morphologic differentiation, progression state at the time of blood sampling or ongoing systemic treatments translated into differences in the abundance of circulating Flt3L, again reminiscent of our observations at the tissue level (illustrated in Figure 4C–G).

We also considered that the circulating levels might reflect tumor independent function of Flt3L as a hematopoietic growth factor, or (tumor-related) comorbidity such as impaired liver or kidney function. However, pathologies in routine laboratory parameters, such as hemoglobin, numbers of leukocytes or thrombocytes, alanine aminotransferase (ALT), gamma-glutamyltransferase (GGT) or creatinine, were not reflected by differences in serum Flt3L (Supplemental Figure S2). Taken together, our observations suggested that Flt3L reflected tumor features that are not well represented by commonly used NEN characteristics.

### 3.4. High Circulating Flt3L Predicts Longer Disease-Related Survival in NEN Patients with Highly Proliferative Tumors

We next explored whether circulating FLT3L indicated the prognosis of NEN patients. The median disease-specific survival in the cohort was 5.9 years, consistent with our focus on cases with highly proliferative advanced stage NEN. ROC analyses indicated 169 pg/mL at 12 months of follow-up as the best cut-off to separate between survivors and patients succumbing to their disease (AUC 0,745).

**Figure 5:**
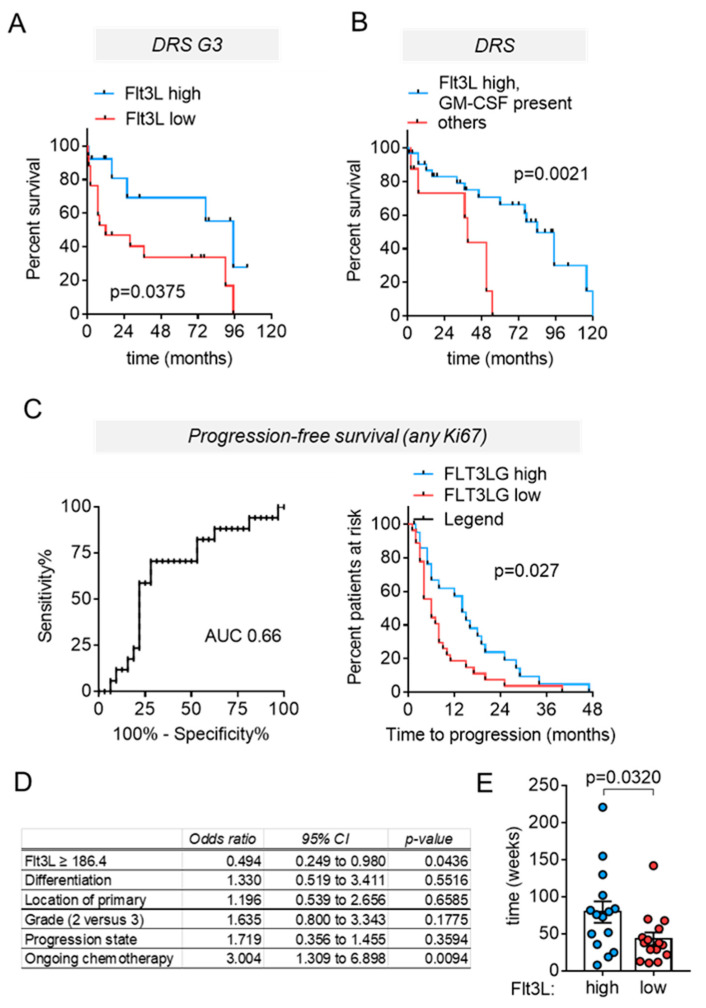
Circulating Flt3L is a prognostic biomarker in NEN. (**A**) Kaplan–Meier curves illustrating disease-related survival of patients with G3 tumors that exhibit above or equal (high) or below (low) cut-off levels of circulating Flt3L (cut-off at 169 pg/mL as determined from ROC curves at 12 months). Median DRS was 95 months in patients with high Flt3L serum levels versus 12 months in patients with low circulating Flt3L (HR 0.3694, 95% CI 0.1466 to 0.9308, logrank test). (**B**) DRS of patients stratified according to Flt3L and GMCSF. Shown are data for the subgroup of patients for which GM-CSF determinations were available (*n* = 40, median survival 76 months). Mean ± SEM GM-CSF was 9.2 ± 2.4 pg/mL, if detectable. Patients with either above mean circulating Flt3L, or detectable GM-CSF, or a combination thereof had better DRS than the remaining patients. Kaplan–Meier estimates of median survival were 95 and 46 months, respectively; HR 0.287, CI 0.0862 to 0.958. (**C**, left panel) ROC curve for circulating Flt3L in patients with or without disease progression at 12 months. (**C**, right panel) Kaplan–Meier estimates of progression-free survival (PFS), indicating median PFS of 14 versus 6 months in patients with circulating FLt3L above or below cut-off (*n* = 22 and 27, respectively, cut-off 186.2 pg/mL; HR 0.557, 95% CI 0.314 to 0.989, logrank test. (**D**) Multivariate Cox regression analysis of PFS. (**E**) Comparison of disease stabilization periods in tumors with Ki67 > 10% following stratification by > mean (high, *n* = 15) or < mean (low, *n* = 15) circulating Flt3L, Mann–Whitney test.

Separation of the entire cohort by above or below cut-off circulating Flt3L levels failed to stratify prognostic subgroups; however, significant differences in disease-related survival became evident when restricting the analysis to samples from patients with G3 tumors (Figure 5A). Moreover, disease-related survival in patients with confirmed tumoral Ki67 fractions >10% differed, when using a survival test that gives more weight to early events, (Supplemental Figure S3A), altogether suggesting the prognostic potential of circulating Flt3L over a period of one year to a few years and/or in patients with more aggressive G3 tumors.

For a subset of our cohort, we had also determined the circulating level of GM-CSF, which supports the recruitment, differentiation, and maturation of cDC1 in a similar fashion to Flt3L [39,40]. Moreover, GM-CSF acts as an endogenous or therapeutic immune-stimulant in solid tumors, in clinical trials and in experimental settings [41]. GM-CSF was below detection limit in 63% of NEN serum samples. The presence of detectable GM-CSF was not associated with NEN characteristics such as grade or stage or with ongoing chemotherapy (not shown). Though Kaplan–Meier estimates failed to demonstrate a significant difference of DRS in patients with or without detectable GM-CSF, survival curves were nonetheless suggestive of a better outcome in patients with detectable levels of the cytokine. Furthermore, GM-CSF levels were not correlated to Flt3L levels, prompting us to combine both cytokines and compare the outcome in patients that had either above cut-off Flt3L levels, or detectable GM-CSF, or both, with patients exhibiting low circulating Flt3L. The combination of both markers allowed an improved prediction of disease-related survival (Figure 5B).

### 3.5. High Circulating Flt3L Predicts Longer Progression–Free-Survival in NEN Patients with Highly Proliferative Tumors

Though disease-related survival represents an important prognostic parameter, estimates of progression free survival (PFS) are more likely to impact on clinical management. ROC curves were constructed at 6 and 12 months (not shown and Figure 5C), indicating 186.4 pg/mL as optimal cut-off based on the Youden’s index. Regression analyses at 6 month intervals confirmed that Flt3L serum levels above mean correlated with PFS at 6 and 12 months, but not at 18 or 24 months after blood sampling. Indeed, the Kaplan–Meier estimates indicated that circulating levels of Flt3L predicted PFS at least as well as grading (Figure 5C and Supplemental Figure S3B). Multivariate Cox regression analyses confirmed circulating Flt3L levels as an independent prognostic factor, whereas tumor differentiation, grading and progression state at sampling had no impact (Figure 5D). Finally, ongoing chemotherapy emerged as an adverse prognostic factor.

### 3.6. High Circulating Flt3L Correlates to Extended Treatment-Induced Disease Stabilization

Almost half of the patients in our cohort experienced disease progression at the time of blood sampling and subsequently received systemic treatment. Therefore, the observed differences in PFS raised the question of whether Flt3L reflected responses to therapy. Unfortunately, few serum samples in our retrospective cohort were exactly matched to treatment start, precluding a straightforward evaluation of Flt3L as a predictive biomarker. As an alternative, we analyzed Flt3L as a mechanistic biomarker, assuming that the Flt3L levels would reflect the response to the preceding treatment. Hence, blood samples were annotated with the time-period from the start of the ongoing or prior treatment until disease progression. By relating Flt3L levels to treatment response in this way, we observed that patients with high circulating Flt3L levels displayed longer disease stabilization periods (Figure 5E). For these analyses, we again included samples from patients with tumoral Ki67 indices > 10% receiving systemic chemotherapies, as this provided a similar distribution of treatment modalities for the sample groups with low and high Flt3L, respectively. A detailed description of the treatment modalities in both groups is provided in Supplemental Figure S4A and the distribution of treatment lines was similar in the two groups (Supplemental Figure S4B). Therefore, high circulating Flt3L was associated with superior PFS following a mixed spectrum of chemotherapies.

## 4. Discussion

Despite the rapidly growing understanding of the molecular features of NEN, treatment decisions in advanced and highly proliferative NEN remain challenging. In part, this may stem from the particularly diverse tumor biology in this subgroup, which encompasses not only prototypic NEC or NET, but also tumors with a long disease and treatment history, giving rise to resistance development and poorly defined dedifferentiation processes. Here, we identified Flt3L, the formative cytokine for cross-presenting dendritic cells, associated with favorable DRS in patients with advanced and highly proliferative NEN across the boundaries of NET or NEC. Prognostic stratification by Flt3L was equally achieved by tissue-based mRNA expression and detection of the circulating cytokine, indicating determination of Flt3L as an easy to implement prognostic test. Within the limitations of a retrospective study in a rare patient subgroup, we furthermore observed an intriguing association of circulating Flt3L with PFS, which potentially reflected an improved response to systemic treatments in patients with high circulating Flt3L levels.

At the tissue level, FLT3LG mRNA expression in G1 NET was exactly within the range of non-transformed pancreas or liver, whereas several of the G2 and G3 tumors exhibited elevated expression levels, which in turn predicted better survival in the Charité cohort. Of note, this cohort was designed to inform on highly proliferative G2 (ki67 ≥ 10%) and G3 NEN, explaining the almost interchangeable median survival times for G2 and G3 subgroups. In addition, the need for snap frozen surgical samples likely biased the clinical features of the G3 cases that were included, favoring early resected localized tumors, less aggressive clinical course, and an overrepresentation of large cell NEC, which are notoriously difficult to distinguish from G3 NET on a purely morphologic basis. Such plausible bias from the surgical procedure, as well as long time remission of individual patients likely account for the long survival in the G3 arm of the cohort. We emphasize, however, that the presence of TP53 and Rb1 mutations supported the morphologic NEC diagnosis.

Our current approach does not allow us to accurately pinpoint the source of Flt3L in these NEN. Based on single cell sequencing data, endothelial cells in the pancreas and T cells in the liver represent potential sources of Flt3L in the healthy organs (data available from V20.1.proteinatlas.org, https://www.proteinatlas.org/ENSG00000090554-FLT3LG/celltype, accessed on 10 June 2021). Within the context of cancer, NK cells, specific T-cell subpopulations and macrophages have been proposed as intratumoral sources [31,42,43]. Our correlative analysis is consistent with T cells or NK cells as Flt3L production sites in NEN. However, supervised clustering of our samples based on the immune cell type specific signatures of the LM22 panel [36] did not reveal any obvious association of Flt3L expression with T or NK cell clusters (not shown). Flt3L production from minor cell populations, or multiple different cell sources may explain this lack of an obvious association. However, high Flt3L mRNA levels were more prevalent in sample clusters with stronger representation of the LM22 signature of activated dendritic cells [36] or a signature of intratumoral dendritic cells [37]. More specifically, we noted a very robust correlation of Flt3L mRNA expression with transcription factors involved in the differentiation and maturation of cross-presenting dendritic cells, suggesting cDC1 as Flt3L targets in the NEN TME. This correlation of Flt3L with specific markers of cDC1 in NEN is remarkable because of the decisive function of this small cell population for the initiation of T cell responses and the development of long-lasting T cell memory [28,44,45]. Importantly, most immunotherapies fail completely in the absence of cross-presenting DC [44,46], which are sparse in many types of tumors, and we and others have shown in preclinical studies that induction of cDC1 at the tumor site using Flt3L can induce response to checkpoint inhibitors in previously unresponsive tumors [30,33]. If the elevated levels of Flt3L observed in our cohort in fact translate to a higher number of cDC1 in the tumor, this could be a strong indicator that the same patients would respond better to checkpoint inhibition than those with low Flt3L expression. If so, this might also explain why first clinical trials with checkpoint inhibitors in NEN failed but significant benefit has been reported in individual patients. Incidentally, one of the patients with high FLT3LG tissue mRNA expression benefited substantially from treatment with a checkpoint inhibitor started 13 months after surgery [22]. In addition, high circulating Flt3L was detected in a blood sample of this patient obtained during the remission phase that followed treatment initiation. These observations agree with a concept of treatment response in individuals with induction of Flt3L, although our retrospective approach, non-matched time points of treatment initiation and blood or tissue sampling as well as a concurrent chemotherapy all preclude a definitive interpretation.

We also observed a correlation between DRS and levels of GM-CSF. GM-CSF is known to promote cDC differentiation and survival and was shown to enhance the early proliferative stages of cDC1 differentiation from CD34+ precursors in vitro [39,40]. Our observation of improved DRS of NEN patients with either detectable GM-CSF or Flt3L could therefore be explained by two complementary pathways, which converge on the functionally relevant shared target of cDC1.

While Flt3L and GM-CSF both appear related to better DRS, there are obvious differences with respect to their production sites in NEN. Flt3L mRNA expression was evident in all NEN tumor samples, whereas the tissue mRNA transcripts for GM-CSF were below the detection limits of our RNAseq approach in 19/22 samples from the high-grade cohort (41/47 in the combined cohort), indicating that circulating GM-CSF reflects its production at extratumoral sites, such as the bone marrow. In contrast, circulating Flt3L may reflect either intratumoral or extratumoral production. Tumoral sources of circulating Flt3L would also explain the lack of correlations between circulating Flt3L and other cytokines from the hematopoietic stem cell panel, except for stem cell factor (SCF) (data not shown, spearman r^2^ = 0.176).

As discussed above, multiple cell types in the TME represent putative Flt3L production sites, including activated T lymphocytes and NK cells [31,42]. Thus, circulating Flt3L levels conceivably reflect—at least in part—effectors of immune control in the TME. Tumor cell intrinsic features in advanced stage disease, hypoxia and metabolic stress or therapy-induced immunogenic cell death represent potential activators of anti-tumor immune responses in our NEN cohort. In fact, most patients of our circulating biomarker cohort had received local or systemic chemotherapies prior to blood sampling, which likely contributed to shaping the immune microenvironment. Chemotherapy-induced immunogenic cell death and the ensuing immune response are thought to account for long-lasting remissions [47,48,49,50]. This concept fits well with our current tentative observation of prolonged treatment-induced disease stabilization in NEN patients with high levels of circulating Flt3L. Precedence for a predictive potential of circulating Flt3L is provided by the recently published results from two studies on oxaliplatin-based chemotherapy, which featured longitudinal measurements of circulating Flt3L as a companion biomarker. In a cohort of patients with high-risk rectal cancer, higher circulating levels of Flt3L were associated with better PFS [51] and in a prospective trial on neoadjuvant hepatic arterial infusion of colorectal cancer liver metastasis, early increases in Flt3L predicted improved recurrence free survival following subsequent surgery [52]. The dynamic increases were interpreted as an indicator of immunogenic cell death in the tumor tissue. It should be noted that many patients in the current NEN cohort had liver tumor burden that far exceeded the tumor burden in the above-cited trial, supporting our notion that circulating Flt3L reflected at least in part its production in the tumor. This would also provide a rationale for Flt3L, but not GM-CSF being predictive for PFS. In addition, tumoral Flt3L expression was identified as a predictive biomarker in a cohort of patients with pancreatic cancer that correlated with improved survival and was independent of other clinical parameters, similar to what we observed in our study [29].

## 5. Conclusions

In conclusion, our data provide a strong rationale for Flt3L as a biomarker of immune response in high-grade NEN. Further prospective studies, especially serial measurements in ongoing clinical trials using immunogenic chemotherapies and/or checkpoint inhibitors in NEN, are needed to confirm the potential of circulating Flt3L to predict responses to (additional) immunotherapies. This could easily be incorporated into the clinical routine to stratify patients and guide therapy decisions in the future.

## Figures and Tables

**Table 1:** Clinical characteristics of the cohort for determination of circulating Flt3L. Characteristics of the serum samples used for determination of circulating Flt3L. Tumor stage refers to AJCC staging criteria. Samples included longitudinal measurements in 9 patients.

			*n* = 59	%
***Age***		Median: 59 Years (Range 29 to 75)		
***Gender***		Female	22	37
		Male	37	63
***Tumor Site***		Pancreas	45	76
		Stomach	10	17
		CUP	4	7
***Tumor Grade***		Grade 2	27	46
		Grade 3 NET	18	30
		Grade 3 NEC	14	24
***Ki67 Index***		<15%	14	24
		≥15%	45	76
***Tumor Stage***		IV	52	88
		I to III	7	12
***Progression State at Blood Sampling***		Complete Remission	7	12
		Stable Disease/Partial Remission	24	41
		Progressive Disease	26	44
***Treatment***		Chemotherapy	11	19
***(At the Time of Blood Sampling)***		Targeted Therapy	2	3
		PRRT	1	2
		None	45	76
***Prior Treatments***	Grade 2	Naïve	8	14
		Pretreated	21	35
	Grade 3	Naïve	8	14
		Pretreated	21	35
		Missing	1	2
***Number of Treatment Modalities*** ***Before Blood Sampling***	Grade 2	Median: 1 (Range 1 to 7)		
	Grade 3	Median: 1 (Range 1 to 5)		
***Type of Treatment***		Streptozotocin/5-FU	11	19
		Temozolomide/Capecitabine	9	15
		FOLFOX	6	10
	Grade 2	Somatostatin Analogs (SSA)	5	8
		Everolimus	3	5
		PRRT	2	3
		SIRT, Brachytherapy, TAE	2	3
		Sunitinib/Bevacizumab	1	2
		Irinotecan	1	2
	Grade 3	FOLFOX	9	15
		Temozolomide/Capecitabine	7	12
		Cisplatin/Etoposide	6	10
		Streptozotocin/5-FU	5	8
		SSA	3	5
		PRRT	3	5
		FOLFIRI	3	5
		Carboplatin/Etoposide	2	3
		SIRT, Brachytherapy, TAE	1	2
		Everolimus	1	2
		Sunitinib/Bevacizumab	1	2
		Capecitabine/Oxaliplatin	1	2
		Carboplatin/Irinotecan	1	2
		Carboplatin	1	2
		Cisplatin	1	2
		Dacarbacin	1	2
		Checkpoint Inhibitor	1	2
		Cisplatin/5-FU/Docetaxel	1	2

**Table 2:** Sample characteristics of the NEN transcriptome cohorts. Description of the samples used for tissue-based analyses of mRNA expression. The combined cohort was used for the correlation of FLT3LG mRNA expression with tumor grade and immune cell markers or signatures. All other tissue-based analyses used data from the Charité subset of samples, thereby minimizing involuntary bias due to different data sources.

			Combined Cohort	%	Charité Cohort	%
***Number of Samples***			*n* = 54	100	*n* = 25	100
***Age***		Median: 64 Years	64 Years		61 Years	
		Range: 32–79 Years	32–79 Years		32–74 Years	
***Sex***		Male	38	70	17	68
		Female	16	30	8	32
***Tumor Site of Origin***		Pancreas	49	91	20	80
		Stomach (All Grade 3)	5	9	5	20
***Tissue Type***	Pimary		38	70	9	36
	Local Recurrence		1	2	1	4
	Metastasis		15	28	15	60
***Tumor Stage (AJCC)***		IA	5	9	0	0
		IB	11	20	1	4
		IIA	5	9	1	4
		IIB	11	20	4	16
		III	1	2	1	4
		IV	21	39	18	72
***Tumor Grade***		G1	14	26	0	0
		G2	19	35	6	24
		G3 NET	12	22	10	40
		G3 NEC	9	17	9	36
***Prior Treatments***		Naïve	N/A		15	60
		Pretreated	N/A		9	36
		Missing	N/A		1	4

## Data Availability

Raw data used in the transcriptome analyses will be made available in the EGA database, EGAD00001006657.

## Supplementary Materials

The following are available online at https://www.mdpi.com/article/10.3390/cancers13174463/s1, Figure S1: Relation of Flt3L mRNA to dendritic cell-specific signatures and ESTIMATE derived overall immune score of NEN transcriptomes; Figure S2: Relation of Flt3L to clinical routine laboratory parameters; Figure S3: Further characterization of circulating Flt3L as a prognostic marker; Figure S4: Comparison of treatment modalities and treatment lines in patients that were included in the analysis of disease stabilization periods; Table S1: Sample characteristics of the Charité tissue cohort.
