# Supplementary material for: Elevated Flt3L Predicts Long-Term Survival in Patients with High-Grade Gastroenteropancreatic Neuroendocrine Neoplasms
Source: Cancers (Basel). 2021 Sep 4;13(17):4463. doi: 10.3390/cancers13174463 (PMC8430927; doi:10.3390/cancers13174463)
Supplement: Supplementary file 1 [file cancers-13-04463-s001.zip › cancers-1330768-SI.pdf]

cancers

MDPI

Article

Elevated Flt3L Predicts Long-Term Survival in Patients with High-Grade Gastroenteropancreatic Neuroendocrine Neoplasms

Katharina M. Detjen [1], Raik Otto [2], Yvonne Giesecke [1], Lukas Geisler [1], Pamela Riemer [3], Henning Jann [1], Carsten Grötzinger [1,6], Christine Sers [3,6], Andreas Pascher [4,†], Tom Lüdde [5], Ulf Leser [2], Bertram Wiedenmann [1], Michael Sigal [1,6,7], Frank Tacke [1], Christoph Roderburg [1,5,*] and Linda Hammerich [1,*]

Table S1. Sample characteristics of the Charité tissue cohort.

			G2	%	G3	%
Number of samples	total n = 25		n = 6	100	n = 19	
Age	(years)	median	47.5		64	
		range	32 - 65		38 -74	
Sex		male	2	8	6	
		female	4	16	13	52
Tumor site of origin		pancreas	6	24	14	56
		stomach	0	0	5	20
Tissue type		pimary	2	8	7	28
		local recurrence	0	0	1	4
		metastasis	4	16	11	44
Tumor stage (AJCC)		IA	0	0		
		IB	1	4		
		IIA	0	0	1	4
		IIB	1	4	3	12
		III	0	0	1	4
		IV	4	16	14	56
Morphology (G3 only)	NET	NET			10 (6)*	40
		prior history of low grade NET			2	8
	NEC	NEC, large cell			3 (1)	12
		NEC, small cell			1	4
		NEC, not specified			5 (2)	20
Prior treatments	naïve		4	16	12	48
	pretreated	SSA	1	4	1	4
		PRRT	2	8	1	4
		Targeted Therapies	1	4	2	8
		STZ/5FU, Tem/Cap	1	4	2	8
		Cis- or Carboplatin/Eto, FOLFOX			3	12
		TACE, SIRT			2	8
		local radiotherapy			1	4

		missing information		1	4
Time from initial diagnosis to tissue sample	NET	median (months)	59.5	15.5	
		range	1-73	<1 - 107	
	NEC (missing data for 1 sample)	median (months)		1.5	
		range		<1 - 32	

* numbers in parenthesis indicate morphologically ambiguous cases, with discrepant classification from routine or study pathologists. Prior history of low grade NET, ki67 fraction > 55%, or evidence of p53, RB1 or K-ras mutations were used to allocate ambiguous G3 samples to either NET or NEC for the purpose of the current study.

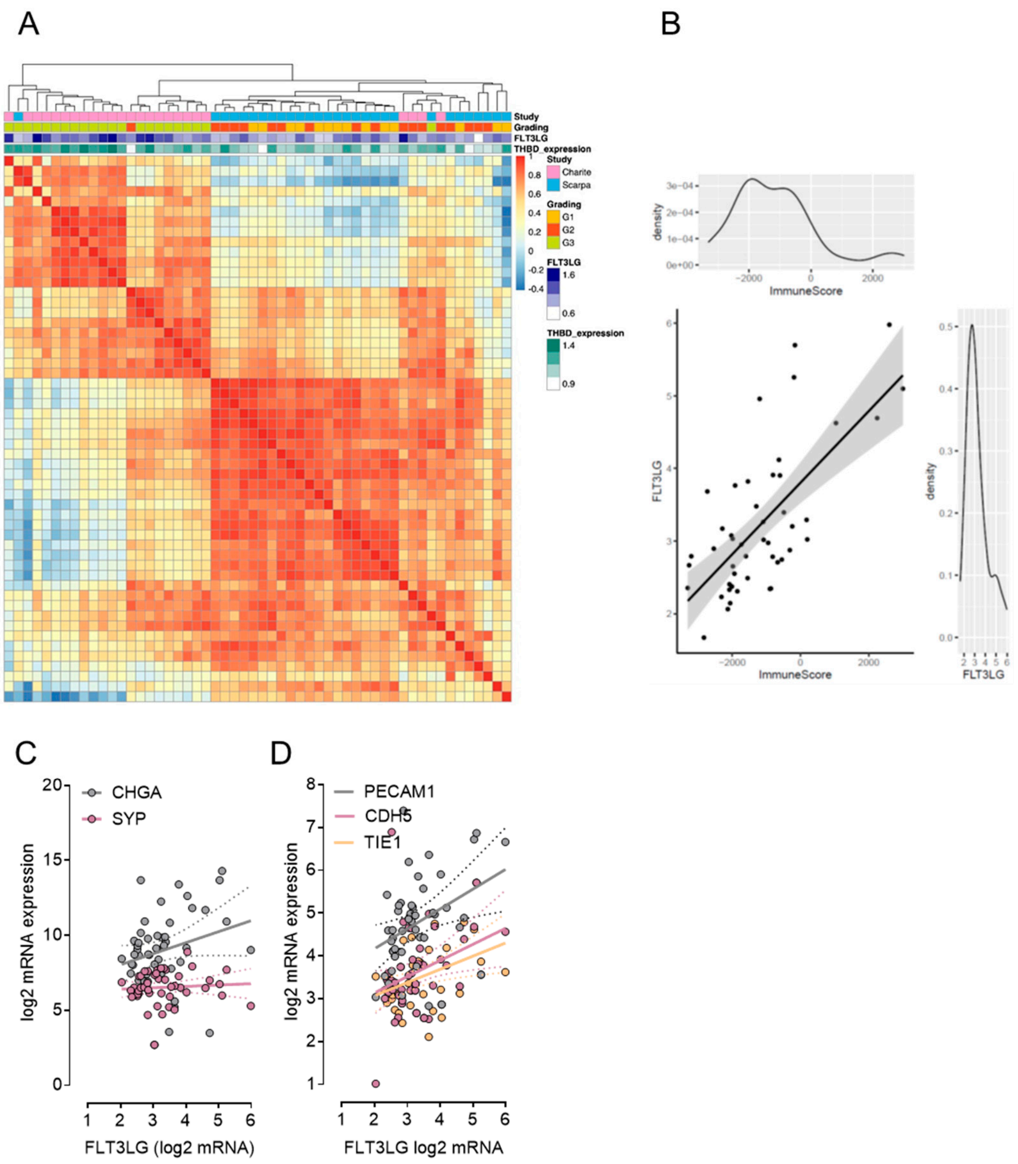

Figure S1. Relation of Flt3L mRNA to dendritic cell-specific signatures and ESTIMATE derived overall immune score of NEN transcriptomes. (A) Heatmap of the correlation-based supervised clustering of samples using a gene signature for intratumoral dendritic cells (Melaiu et al. DOI:

10.1038/s41467-020-19781-y). Additional annotations are provided for grading, FLT3LG expression and average expression of the signature genes (referred to as THBD_expression). (B) Scatter plot illustrating the relation of FLT3LG expression to the immune score as determined by the ESTIMATE (Estimation of STromal and Immune cells in MAlignant Tumours using Expression data algorithm (Yoshihara et al. DOI: 10.1038/ncomms3612). Despite the overall good correlation, samples with above mean Flt3L mRNA expression exhibit a wide range of immune scores, suggesting that Flt3L mRNA expression can be separated from a high overall immune cell content of the tumor in individual samples. (C) Lack of correlation between FLT3LG and neuroendocrine differentiation markers CHGA (rs = .26, p =0.0699, n=49) or SYP (rs = - 0.17, p = 0.24, n=49). (D) Missing (TIE1, rs 0.19, p= 0.188; CDH5, rs 0.27, p=0.059) or weak (PECAM1, rs = 0.349, p=0.013) correlation of FLT3LG mRNA to markers of endothelial cells expressed in all samples (n=49).

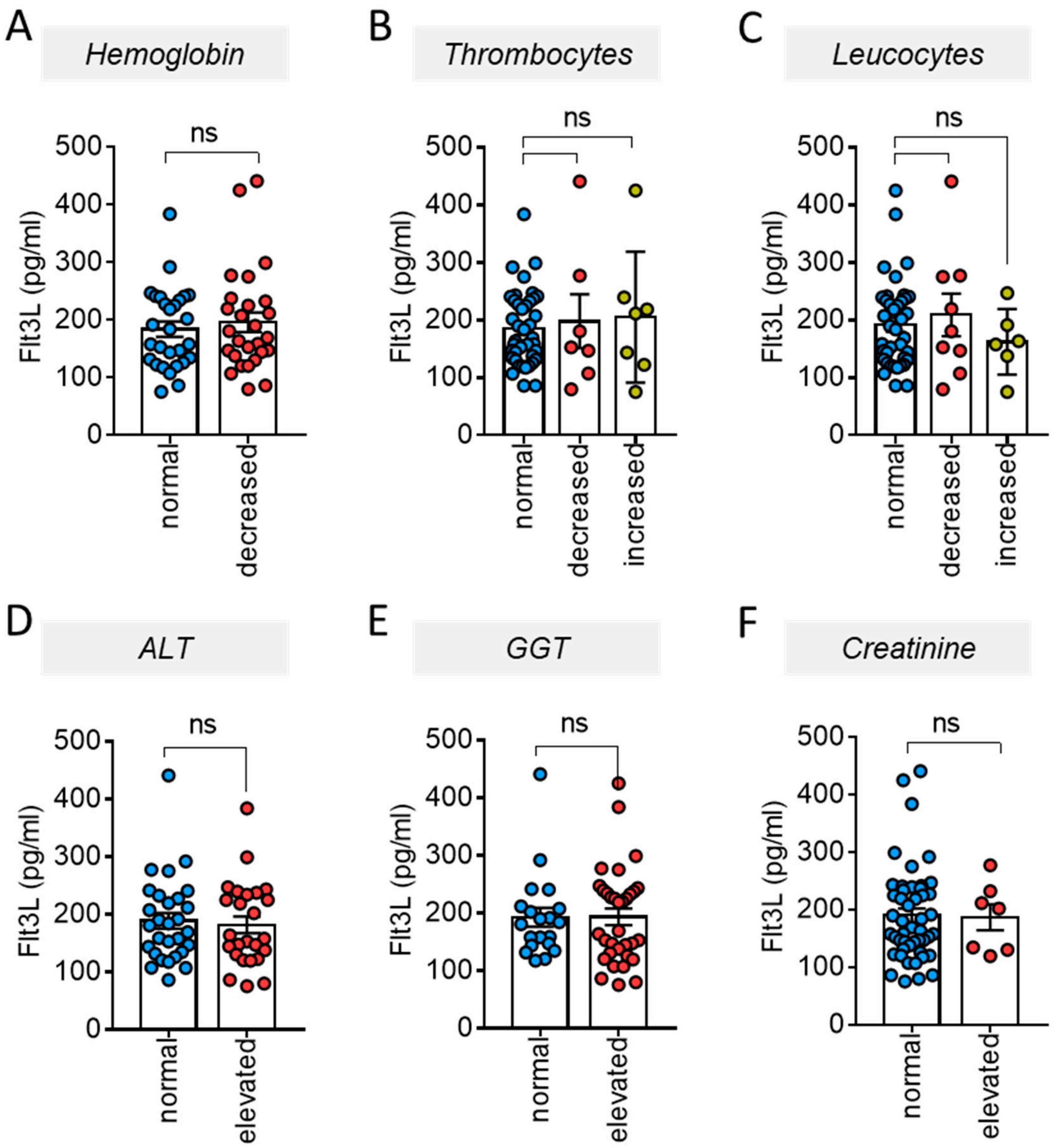

Figure S2. Relation of Flt3L to clinical routine laboratory parameters**.** Flt3L levels in sera from patients with pathologic levels of clinical routine blood laboratory parameters hemoglobin ***(A)***, numbers of thrombocytes (***B)*** or leucocytes ***(C),*** alanine aminotransferase (ALT) ***(D)***, gamma-glutamyltransferase (GGT) ***(E)*** or creatinine ***(F)*** were not different from those with normal measurements for these parameters. Bars and error bars indicate Mean± SEM. Mann-Whitney (A, D-E) and Kruskall-Wallis test (B, C) were used for determination of p-values; p>0.05 was considered not significant (ns).

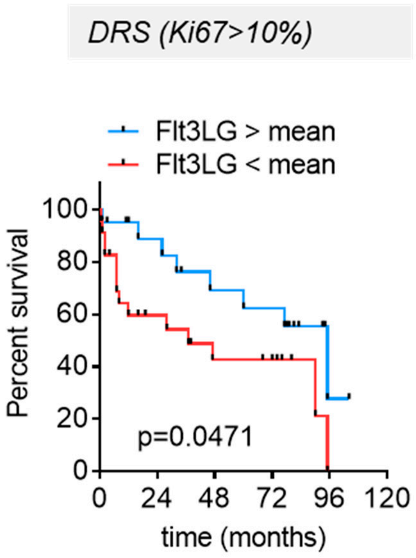

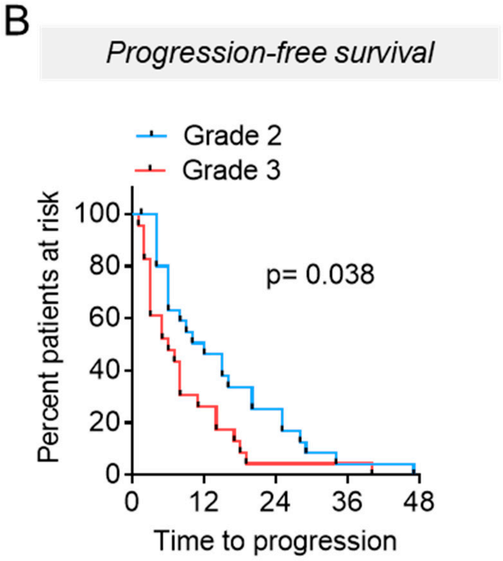

Figure S3. Further characterization of circulating Flt3L as a prognostic marker. (**A**) Kaplan Meier curves illustrating disease-related survival of patients with G3 tumors that exhibit above or equal (high) or below (low) cut-off levels of circulating Flt3L (cut-off at 169 pg/ml as determined from ROC curves at 12 months). Significant differences in survival as indicated by the p-value were observed using the Gehan-Breslow-Wilcoxon test, which gives more weight to earlier time points. Analysis by Log-rank test barely missed significance, p= 0.06. Median DRS was 95 months in patients with high Flt3L serum levels versus 37 months in patients with low circulating Flt3L (HR 0.39, 95%CI 0.163 to 0.928). (**B**) Performance of tumor grading as prognostic indicator of progression-free survival of NEN. Median PFS was 12 and 6 months in G2 and G3 tumors, respectively. HR 0.547, 95%CI 0.2996 to 0.9985.

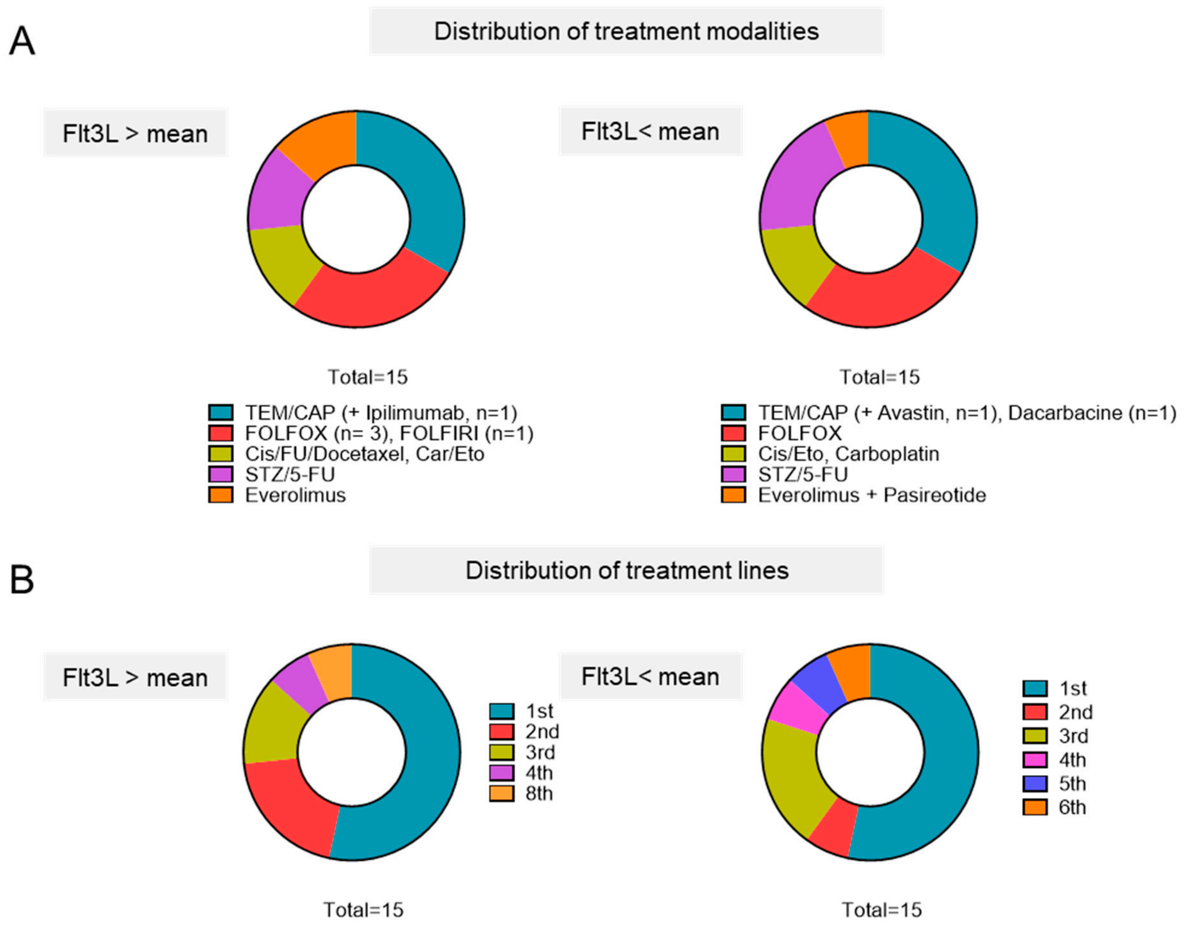

Figure S4. Comparison of treatment modalities and treatment lines in patients that were included in the analysis of disease stabilization periods (as shown in Manuscript Figure 5D) (A) illustration of the different treatment modalities that were applied around the time or prior to blood sampling for Flt3L determinations. Treatment modalities attest to individual clinical management with respect to targeted drugs, but also to an overall similar distribution of major treatment groups (Tem/Cap; FOLFOX). (B) Illustration of treatment lines. More than half of the patients in each group received first line systemic therapies, and 2nd plus 3rd line treatments accounted for another 25 to 30%. Abbreviations are Temozolomide (TEM), Capecitabine (CAP), Cisplatin (Cis), Carboplatin (Car), Etoposide (Eto), Streptozotocine (STZ).
